# Five years after the pandemic: rethinking the pharmacological management of mental disorders

**DOI:** 10.3389/fpsyt.2025.1750073

**Published:** 2026-01-12

**Authors:** Esteban Zavaleta-Monestel, Ricardo Millán-González, Jeaustin Mora-Jiménez, Sebastián Arguedas-Chacón

**Affiliations:** 1 Health Research Department, Clínica Bíblica, San José, Costa Rica; 2 Department of Pharmacy, Clínica Bíblica, San José, Costa Rica; 3 Faculty of Medicine, Universidad de Costa Rica, San José, Costa Rica; 4 Department of Psychiatry, Clínica Bíblica, San José, Costa Rica

**Keywords:** COVID-19, digital mental health, pharmacovigilance, precision psychiatry, psychopharmacology, substance use, telepsychiatry

## Introduction

The COVID-19 pandemic profoundly altered the landscape of global mental health. Early meta-analyses revealed a sharp rise in anxiety, depressive symptoms, and insomnia across populations, with pooled prevalence rates exceeding 30% for both depression and anxiety (1). Subsequent longitudinal reviews reported that such disturbances often persisted months after infection or lockdown measures, indicating a sustained burden on psychological well-being (2).

Parallel to this epidemiologic shift, patterns of psychotropic medication use changed worldwide. Systematic evidence indicates that the pandemic disrupted prescribing practices, modifying both the classes and volumes of agents dispensed (3). Multinational network analyses later reported sustained increases in prescriptions for antidepressants, anxiolytics, and sedatives even as restrictions lifted. At the same time, similar trends were observed among children and adolescents, underscoring the breadth of this pharmacologic response (4, 5).

Emerging evidence indicates that post-traumatic and substance-use–related conditions significantly intensified the psychiatric burden of the COVID-19 era. Meta-analytic data report early prevalence rates of post-traumatic stress disorder (PTSD) up to 43% and anxiety up to 46%, accompanied by depressive symptoms, cognitive disturbances, and sleep disorders across hospitalized and community cohorts. Vulnerability was especially pronounced among healthcare workers and individuals with pre-existing substance use disorders (SUDs). In parallel, increased substance use (SU), particularly alcohol, tobacco, or nicotine, and other legal and illicit substances, was frequently reported as a coping response to pandemic-related stress and psychiatric symptoms. Together, these intertwined trajectories of PTSD, anxiety, and escalated SU had a measurable impact on psychotropic prescribing patterns and should be integrated into post-pandemic pharmacotherapy analyses (6).

Beyond behavioral drivers, emerging biological data suggest that the pandemic’s legacy extends to neuroimmune and inflammatory mechanisms that may influence treatment response. Neuroimaging and biomarker studies have identified persistent microglial activation and gliosis in individuals with post-COVID neuropsychiatric symptoms. At the same time, integrative reviews propose that systemic inflammation may contribute to depressive and cognitive sequelae. These insights converge with broader evidence linking dysregulated cytokine and immune pathways to major mood disorders, inviting cautious reconsideration of pharmacologic targets within psychiatry (7, 8).

Rather than viewing the pandemic as a transient interruption to established systems of care, the accumulated experience offers an opportunity to examine how crisis-driven adaptations, spanning access, prescribing practices, and mechanistic research, have reshaped psychiatric pharmacotherapy. Synthesizing these lessons is essential to inform a more rational, equitable, and biologically informed therapeutic approach for the years ahead.

## Disrupted systems and adaptive prescribing

The pandemic precipitated an unprecedented restructuring of psychiatric service delivery worldwide. Telepsychiatry rapidly became a principal channel for patient contact, with mental-health-related virtual visits increasing more than tenfold compared with pre-pandemic levels and remaining substantially elevated through 2022 (9). Emergency regulatory waivers facilitated this expansion: temporary federal provisions permitted remote prescribing of controlled medications, thereby maintaining treatment continuity when in-person evaluations were restricted (10).

While these policy adjustments safeguarded access, they also introduced variability in medication adherence and equity. Multi-system cohort analyses revealed that, although overall adherence to antidepressant and antipsychotic therapy sometimes improved, significant racial and socioeconomic disparities persisted (3). Concomitantly, prescribing patterns shifted across therapeutic classes. A global systematic review documented surges in antidepressant, anxiolytic, and hypnotic use, whereas antipsychotic long-acting injectables experienced early declines due to service interruptions (11). Notably, multinational network data later showed that altered prescription volumes persisted even after restrictions eased, indicating structural rather than transient changes in practice (12).

Health systems and patients adapted through digital and logistical innovations. The introduction of online and pharmacy models enabled medication renewal and delivery when clinics were closed, helping mitigate supply-chain disruptions. Yet access to these tools was uneven: systematic evidence shows disparities in telehealth and e-pharmacy uptake across rural, older, and minority populations (13).

Collectively, these experiences revealed both the resilience and fragility of modern psychiatric pharmacotherapy systems. Rapid innovation preserved care during the crisis but underscored the need for robust oversight, equitable digital infrastructure, and rational prescribing frameworks as psychiatry transitions toward hybrid models of medication management.

## The biological legacy of the pandemic

SARS-CoV-2 can influence the central nervous system through neuroimmune pathways relevant to mood and cognition, providing a plausible biological substrate for post-infection psychiatric symptoms (14). Neuroimaging studies using translocator-protein positron emission tomography (TSPO PET) have reported elevated glial activation in individuals with persistent post-COVID neuropsychiatric manifestations. These findings are consistent with neuroinflammatory processes that may modulate affective and cognitive function (7). However, these observations should be interpreted cautiously, as reported group differences are modest, show substantial overlap, and are based on relatively small samples, reflecting the exploratory nature of current biomarker research. More broadly, immunopsychiatric literature demonstrates dysregulated cytokine signaling, particularly interleukin-6 and tumor necrosis factor α, across mood disorders, suggesting overlapping inflammatory pathways that may interact with pandemic-related stressors rather than constituting disease-specific mechanisms (8).

Chronic psychosocial stress during and after the pandemic has also been linked to hypothalamic–pituitary–adrenal-axis dysregulation and immune activation, reinforcing established connections between stress, inflammation, and depressive symptomatology (15). In parallel, extensive cohort analyses indicate elevated incident risks of multiple mental-health outcomes following SARS-CoV-2 infection, supporting the relevance of biological vulnerability alongside psychosocial factors in post-pandemic psychiatric burden (16).

From a therapeutic perspective, meta-analytic data suggest that adjunctive anti-inflammatory agents can reduce depressive symptoms in selected patient subgroups, although substantial heterogeneity, modest effect sizes, and publication bias limit broad generalization (17). In parallel, rapid-acting glutamatergic interventions such as ketamine and esketamine demonstrate the potential of treatments that act beyond traditional monoaminergic mechanisms and highlight emerging efforts to align pharmacotherapy with biologically informed stratification strategies (18). Comprehensive reviews of post-acute sequelae of COVID-19 further emphasize persistent neuroinflammatory signals and their possible implications for treatment response, underscoring the need for cautious, multidisciplinary, and hypothesis-driven integration of biological markers into post-pandemic psychiatric practice rather than premature clinical application (19).

## Between innovation and overprescription

The pandemic accelerated the adoption of new pharmacologic and care-delivery approaches, but it simultaneously magnified risks of medication overuse and fragmented prescribing. Systematic reviews documented marked shifts in psychotropic prescribing across antidepressants, anxiolytics, and hypnotics, reflecting adaptive yet sometimes uncoordinated responses to widespread psychosocial stressors (3). Gender-stratified analyses from the United States identified early increases in benzodiazepine and Z-hypnotic use, particularly among women, followed by partial declines. At the same time, serotonergic antidepressant prescribing remained elevated around the onset of the pandemic (20). Among older adults, population-based studies showed higher post-discharge initiation of antipsychotics and benzodiazepines during pandemic peaks, underscoring heightened vulnerability to inappropriate or excessive prescribing in this group (21).

Telemedicine-based prescribing by mental health clinicians became increasingly common, expanding access to care while also raising concerns regarding diagnostic accuracy, continuity, and clinical oversight in remote settings (22). In parallel, app-based digital interventions demonstrated small-to-moderate improvements in depressive symptoms in controlled trials, suggesting potential benefit when used as adjuncts rather than stand-alone treatments (23). Concurrently, umbrella reviews indicate that pharmacogenomic-guided treatment may improve outcomes for selected patients, supporting a cautious role for biomarker-informed prescribing within clearly defined clinical contexts. Complementary deprescribing frameworks have also emerged to address cumulative medication burden and to guide safe, structured tapering strategies (24).

Maintaining balance between innovation and stewardship remains essential. Consensus experts emphasize that progress in psychiatry depends on integrating technological and biological advances with evidence-based prescribing principles, deprescribing where appropriate, and sustained attention to person-centered care (25). Without such integration, there is a risk that new tools and delivery models may replace earlier forms of therapeutic fragmentation rather than meaningfully improving long-term outcomes.

## Toward a post-pandemic framework for rational pharmacotherapy

The pandemic exposed critical gaps in medication-safety systems and reinforced the need for strengthened pharmacovigilance, as highlighted by World Health Organization reports documenting uneven adverse-event reporting (26). Integrated-care models have demonstrated improvements in adherence, reductions in emergency visits, and fewer adverse events, supporting their relevance to more rational approaches to pharmacotherapy in the post-pandemic context (27). Digital health tools, including smartphone-based interventions and web platforms, can provide additional support for symptom monitoring and prescribing decisions. At the same time, pharmacogenomic and biomarker-based strategies may enhance precision in antidepressant selection and dosing for selected patients (28). Persistent inequities in access to mental health treatments underscore the importance of policies that promote affordability, digital inclusion, and system-level equity (29). Updated deprescribing guidelines and national surveillance data further illustrate ongoing shifts in prescribing patterns and highlight the continued importance of post-marketing oversight. Taken together, these developments help inform key elements of a future-oriented approach to psychopharmacology grounded in safety, personalization, and equity, rather than defining a single, uniform model of care (30).

The post-pandemic expansion of digital health also highlights the growing yet still evolving role of artificial intelligence in pharmacovigilance. Machine-learning systems, including co-clustering algorithms and transformer-based language models, can analyze large-scale clinical datasets, prescription records, and unstructured sources such as social media to support earlier detection of potential safety signals compared with traditional methods. Emerging evaluations suggest that AI-based tools may improve adverse-event extraction, accelerate signal detection, and enhance monitoring of drug–drug interactions and substance-use patterns, particularly in populations with complex mental health and comorbidity profiles. When integrated cautiously with established surveillance networks and clinical governance structures, AI-assisted pharmacovigilance has the potential to contribute to more proactive and scalable medication-safety monitoring, while requiring ongoing validation and oversight (31, 32).

## Discussion

Five years after the pandemic, psychiatric pharmacotherapy is navigating an evolving landscape shaped by innovation, safety considerations, and ongoing system redesign. Global reports from the World Health Organization and the OECD underscore that the pandemic both intensified pre-existing mental health burdens and exposed persistent weaknesses in pharmacovigilance and access (33, 34). The WHO Global Patient Safety Report 2024 identifies medication-related harm as a major preventable cause of morbidity, emphasizing the need for stronger post-marketing surveillance and reporting systems. These observations align with extensive pharmacoepidemiologic analyses demonstrating sustained increases and class shifts in psychotropic prescribing since 2020, indicating that crisis-driven practices have produced lasting structural effects on medication use (30).

The expansion of telepsychiatry and digital mental health tools remains one of the most durable transformations in clinical practice. Systematic reviews indicate that remote and blended care models can achieve clinical effectiveness comparable to in-person visits, while meta-analyses of digital interventions report consistent, though generally modest, benefits for depressive symptoms (35). However, underreporting of adverse events and variable data privacy standards highlight the need for stronger governance frameworks (36). Together, these mixed findings suggest a field in transition, moving from emergency-driven improvisation toward more regulated and evidence-based digital integration.

Parallel developments in personalized and precision psychiatry have begun to influence therapeutic decision-making. Updated umbrella reviews confirm that pharmacogenomic-guided antidepressant selection can improve response and tolerability in selected patients, although effect sizes remain moderate and population-level implementation is uneven. Emerging neuroimaging studies linking brain-circuit biomarkers to treatment response point toward future biologically informed stratification strategies, yet experts consistently caution that translation into routine practice will require further replication, cost-effectiveness analyses, and clinician training (37). Collectively, these data support cautious optimism, recognizing meaningful progress without overstating readiness for widespread clinical deployment (38).

Alongside these scientific advances, renewed efforts to redesign care delivery have gained prominence. International reviews of collaborative care models consistently demonstrate improvements in adherence, symptom reduction, and reduced emergency service utilization compared with fragmented care (39, 40). By embedding pharmacotherapy within multidisciplinary teams, these models align medication management with psychological and social interventions. However, the OECD’s 2025 analysis of mental health inequalities indicates that access to such integrative services remains uneven, particularly for rural, older, and lower-income populations, reinforcing equity as a central determinant of post-pandemic recovery (41).

Taken together, current evidence suggests that psychiatry is entering a phase of consolidation rather than transformation. The acute disruptions of the pandemic accelerated experimentation with digital prescribing, remote monitoring, and biomarker-informed care, but also amplified risks of overprescription, inequity, and inconsistent oversight. The challenge now lies in translating these advances into more coherent, safe, and equitable pharmacotherapeutic practices. Consensus experts emphasize the importance of coupling real-world data monitoring with rational prescribing and deprescribing strategies, embedding pharmacovigilance and patient participation across the continuum of care (42). Aligning innovation with stewardship is therefore essential to ensure that post-pandemic adaptations mature into sustainable, evidence-based improvements.

**Figure 1:**
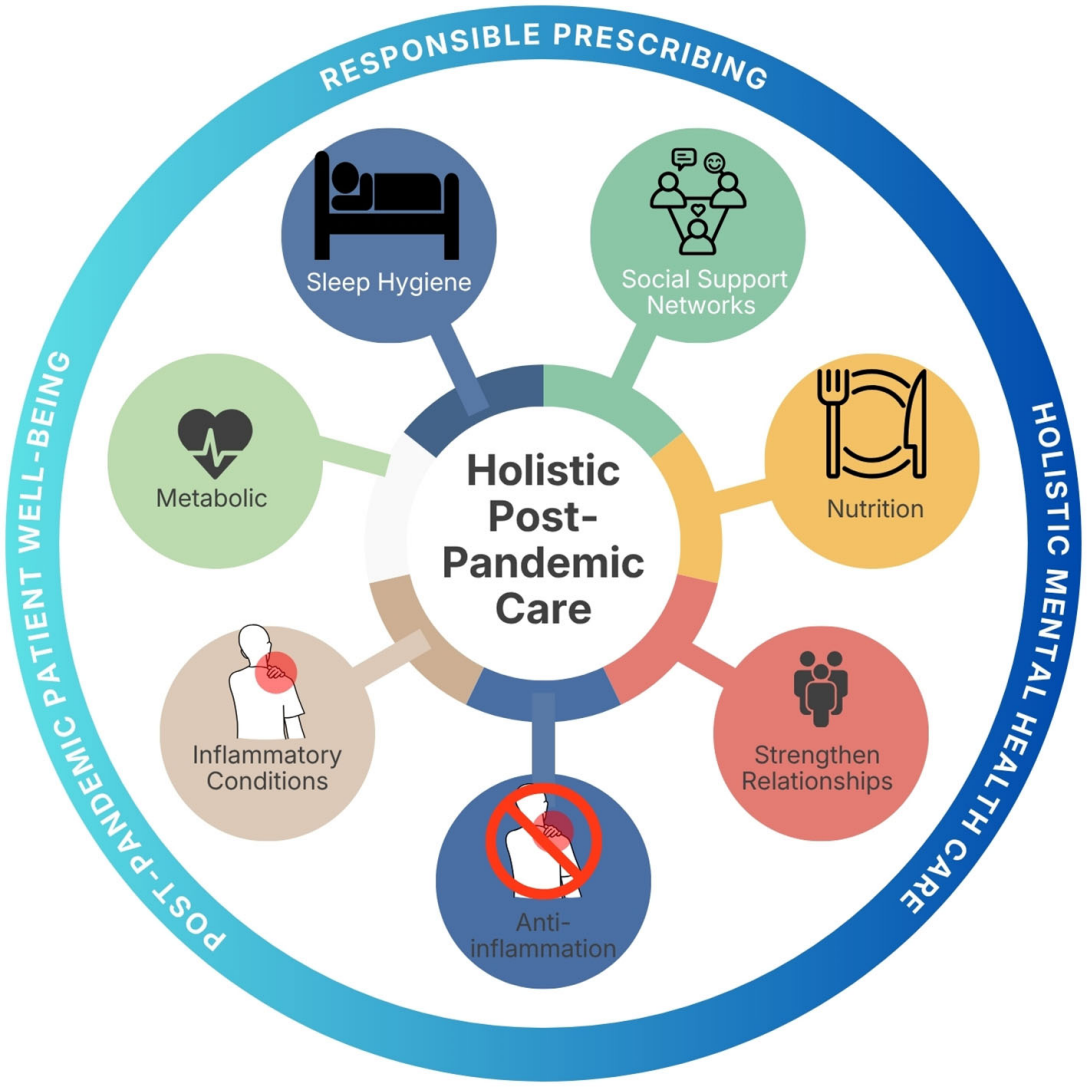
Holistic framework for post-pandemic mental health care.

Responsible prescribing must also be grounded in a holistic understanding of patient well-being. Non-pharmacological strategies, including sleep hygiene, structured physical activity, nutrition, strengthening social support networks, and addressing underlying metabolic or inflammatory conditions, remain integral to comprehensive mental health care. Integrating these approaches alongside pharmacotherapy reinforces a balanced, person-centered model of care. As illustrated in **Figure 1**, these domains collectively represent the core components of a holistic post-pandemic approach to mental health management.

In summary, the pandemic catalyzed rapid innovation in telepsychiatry, digital prescribing, pharmacogenomics, and biomarker research, expanding therapeutic possibilities while simultaneously revealing enduring gaps in safety, equity, and coherence. Rather than marking a definitive turning point, this period offers an opportunity to consolidate lessons learned and refine existing tools. Progress in the coming decade will depend less on the introduction of new medication classes than on more thoughtful, safer, and more equitable use of those already available, thereby transforming crisis-driven adaptation into durable progress for global mental health.
